# Drug-induced hypersensitivity syndrome caused by minodronic acid hydrate

**DOI:** 10.1186/s12890-021-01709-x

**Published:** 2021-11-07

**Authors:** Yutaka Muto, Naoyuki Kuse, Minoru Inomata, Nobuyasu Awano, Mari Tone, Kohei Takada, Kazushi Fujimoto, Yuan Bae, Toshio Kumasaka, Takehiro Izumo

**Affiliations:** 1 Department of Respiratory Medicine, Japanese Red Cross Medical Center, 4-1-22 Hiroo, Shibuya-ku, Tokyo, 150-8953 Japan; 2 Department of Pathology, Japanese Red Cross Medical Center, 4-1-22 Hiroo, Shibuya-ku, Tokyo, 150-8953 Japan

**Keywords:** Drug-induced hypersensitivity syndrome, Drug reaction with eosinophilia and systemic symptoms, Interstitial lung disease, Cryobiopsy, Case report

## Abstract

**Background:**

Drug-induced hypersensitivity syndrome (DIHS)/drug reaction with eosinophilia and systemic symptoms (DRESS) syndrome is an important adverse reaction caused by a few drugs. Reactivation of human herpesvirus 6 (HHV-6) is known to be associated with its pathogenesis. DIHS occasionally manifests as pulmonary lesions with a variety of imaging findings.

**Case presentation:**

An 83-year-old woman started taking minodronic acid hydrate 5 years before admission. She noticed a generalized skin rash 44 days before admission and started oral betamethasone-*d*-chlorpheniramine maleate combination tablets for allergic dermatitis. She developed a fever and cough in addition to the rash, and was referred to our hospital. Laboratory data showed a high level of eosinophils and liver and biliary enzymes. Computed tomography (CT) studies revealed bilateral diffuse ground-glass opacities with ill-defined centrilobular nodules from the central to peripheral regions of the lungs. Transbronchial lung cryobiopsy specimens showed that lymphocyte infiltration was observed in the alveolar walls and fibrinous exudates and floating macrophages in the alveolar lumina. Immunohistochemistry of biopsy specimens showed more CD4^+^ lymphocytes than CD8^+^ lymphocytes, while few Foxp3^+^ lymphocytes were recognized. The serum anti-HHV-6 immunoglobulin G titer increased at 3 weeks after the first test. Based on these findings, we diagnosed her with DIHS. We continued care without using corticosteroids since there was no worsening of breathing or skin condition. Eventually, her clinical symptoms chest CT had improved. Minodronic acid hydrate was identified as the culprit drug based on the positive results of the patch test and drug-induced lymphocyte stimulation test.

**Conclusions:**

We described the first case of DIHS caused by minodronic acid hydrate. Lung lesions in DIHS can present with bilateral diffuse ground-glass opacities and ill-defined centrilobular nodules on a CT scan during the recovery phase. Clinicians should be aware of DIHS, even if patients are not involved with typical DIHS/DRESS-causing drugs.

**Supplementary Information:**

The online version contains supplementary material available at 10.1186/s12890-021-01709-x.

## Background

Drug-induced hypersensitivity syndrome (DIHS)/drug reaction with eosinophilia and systemic symptoms (DRESS) syndrome is an adverse drug reaction associated with the sequential reactivation of human herpes virus-6 (HHV-6). It is caused by a small number of drugs, such as anticonvulsants, allopurinol, and sulfonamides [1]. The symptoms are characterized by rash, fever, lymphadenopathy, hepatitis, and leukocytosis with eosinophilia. DIHS/DRESS causes multiple organ damage, with a mortality rate of 10% [2]. Pulmonary lesions are uncommon and are recognized in 5% of DIHS/DRESS cases [2]. The common pulmonary manifestation on computed tomography (CT) findings of DIHS/DRESS is the interstitial infiltrates. However, bilateral nodules, lobular central granular shadows, and pleural effusions were also reported [1–5]. We here describe the first case of DIHS/DRESS caused by minodronic acid hydrate accompanied by diffuse ground-glass opacities with ill-defined centrilobular granular nodules on a chest CT scan.

## Case presentation

An 83-year-old woman started taking 50 mg of minodronic acid hydrate monthly 5 years before admission because of osteoporosis. She had regularly been taking some additional medications, such as aspirin, clopidogrel sulfate, and rosuvastatin calcium. She developed itchy erythematous papules 44 days before admission. Her family doctor suspected clopidogrel-induced skin rash. She was ordered to stop the drug and prescribed a betamethasone-*d*-chlorpheniramine maleate combination agent. Despite the treatment with anti-allergic medicine, she developed a fever and dry cough in addition to the persistent rash. Then, she was referred to our hospital for additional evaluation.

**Fig. 1:**
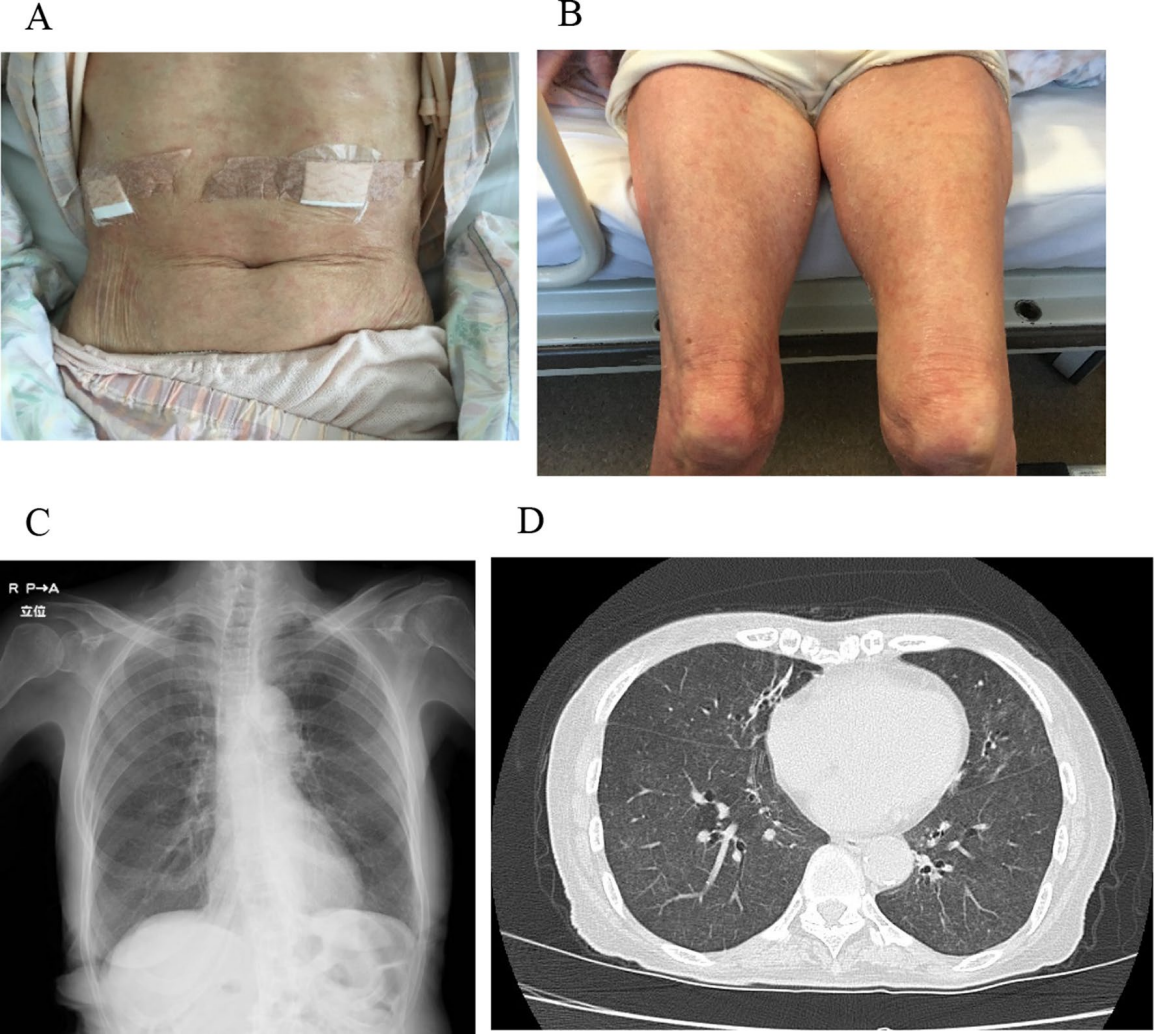
Skin lesions and chest images. **A**, **B** Erythematous papules with desquamation are recognized on the trunk and limbs. **C** Chest X-ray shows bilaterally diffuse granular shadows. **D** Ground-glass opacities and ill-defined centrilobular nodules are revealed from the central to peripheral regions of the lungs on chest computed tomography

**Fig. 2:**
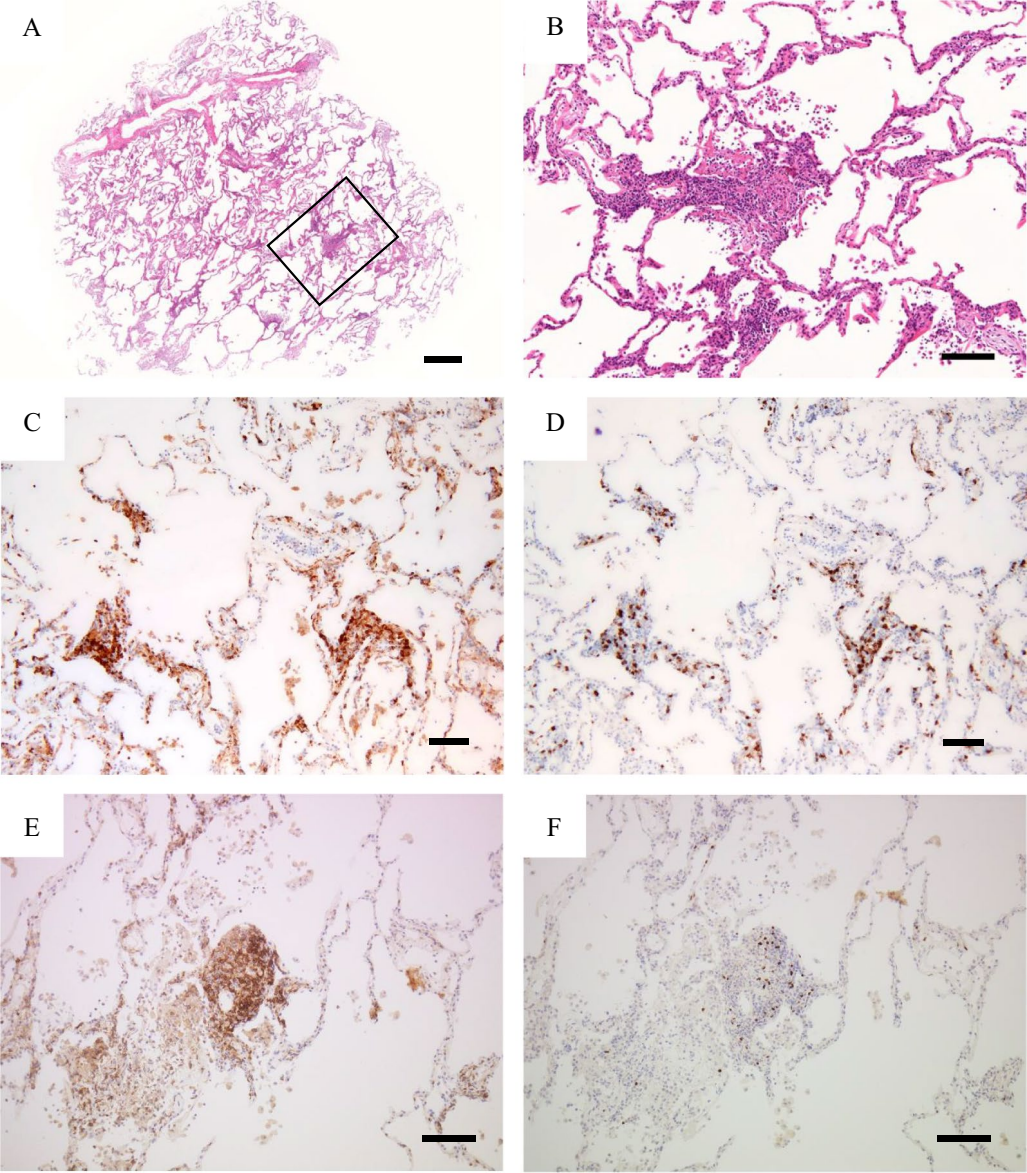
Pathological findings of lung biopsy specimens. **A** Lymphocytes and hyperplasia of type II pneumocytes infiltrate the alveolar walls (hematoxylin and eosin staining, ×40). **B** A high magnification of an area of a square of **A** shows few eosinophils. Fibrinous exudates and floating macrophages are observed in the alveolar lumina (hematoxylin and eosin staining, ×200). **C**, **D** Immunohistochemistry of biopsy specimens with CD4 (**C**) and CD8 (**D**) stains shows more CD4^+^ lymphocytes than CD8^+^ lymphocytes (×100). **E**, **F** Immunohistochemistry of biopsy specimen with CD4 (**E**) and Foxp3 (**F**) stains shows few CD4^+^ Foxp3^+^ lymphocytes (×100). Scale bar = 500 µm in (**A**), 50 µm in (**B**), and 100 µm in (**C**, **F**). The sections were observed with a microscope: Axio Scope A1 (Zeiss, Oberkochen, Germany), lenses: Plan-Apo × 5, 10, 20 (Nikon, Tokyo, Japan), a camera: FLOVEL Model FR-630 M (FLOVEL CO, LTD, Tokyo Japan), and a photo system: FLOVEL Image Filling system (FLOVEL CO, LTD, Tokyo, Japan)

On hospital admission, her vital signs were as follows: a body temperature of 36.9 °C on acetaminophen, respiratory rate of 17/min, and percutaneous oxygen saturation (SpO_2_) of 97% (ambient room air). On physical examination, chest auscultation revealed no crackles. Palpitation revealed bilateral inguinal lymphadenopathy and slow pitting edema on both legs. Erythematous papules with desquamation were recognized on the trunk and limbs (Fig. 1A, B). Laboratory investigations showed elevation of the white blood cell count (8030/μL) with eosinophilia (1090/μL) with the administration of betamethasone-*d*-chlorpheniramine maleate combination agent and high immunoglobulin E (IgE) levels (240 U/mL). Liver and biliary enzymes were also elevated. Chest radiography showed bilaterally diffuse granular shadows (Fig. 1C). CT scan of the chest demonstrated bilateral diffuse ground-glass opacities with ill-defined centrilobular nodules from the central to peripheral regions of the lungs. (Fig. 1D). Bronchoalveolar lavage fluid (BALF) revealed inflammatory changes with a cell differential count of 40% macrophages, 58% lymphocytes, and 2% neutrophils, and the CD4/CD8 ratio was elevated at 6.2. Transbronchial lung cryobiopsy (TBLC) was performed in the left lower lobe. Histological examination revealed infiltration of lymphocytes in the alveolar walls and hyperplasia of type II pneumocytes (Fig. 2A). Few eosinophils were recognized. Fibrinous exudates and floating macrophages were observed in the alveolar lumina (Fig. 2B). Immunohistochemistry of biopsy specimens with CD4 and CD8 staining showed more CD4^+^ T cells than CD8^+^ T cells (Fig. 2C, D, Additional file 1: Figure S1A, S1B). Among the CD4^+^ T cells, there were few Foxp3^+^ T cells (Fig. 2E, F, and Additional file 1: Figure S1C). Two abdominal skin lesions were biopsied; they showed light perivascular infiltration of lymphocytes.

**Fig. 3:**
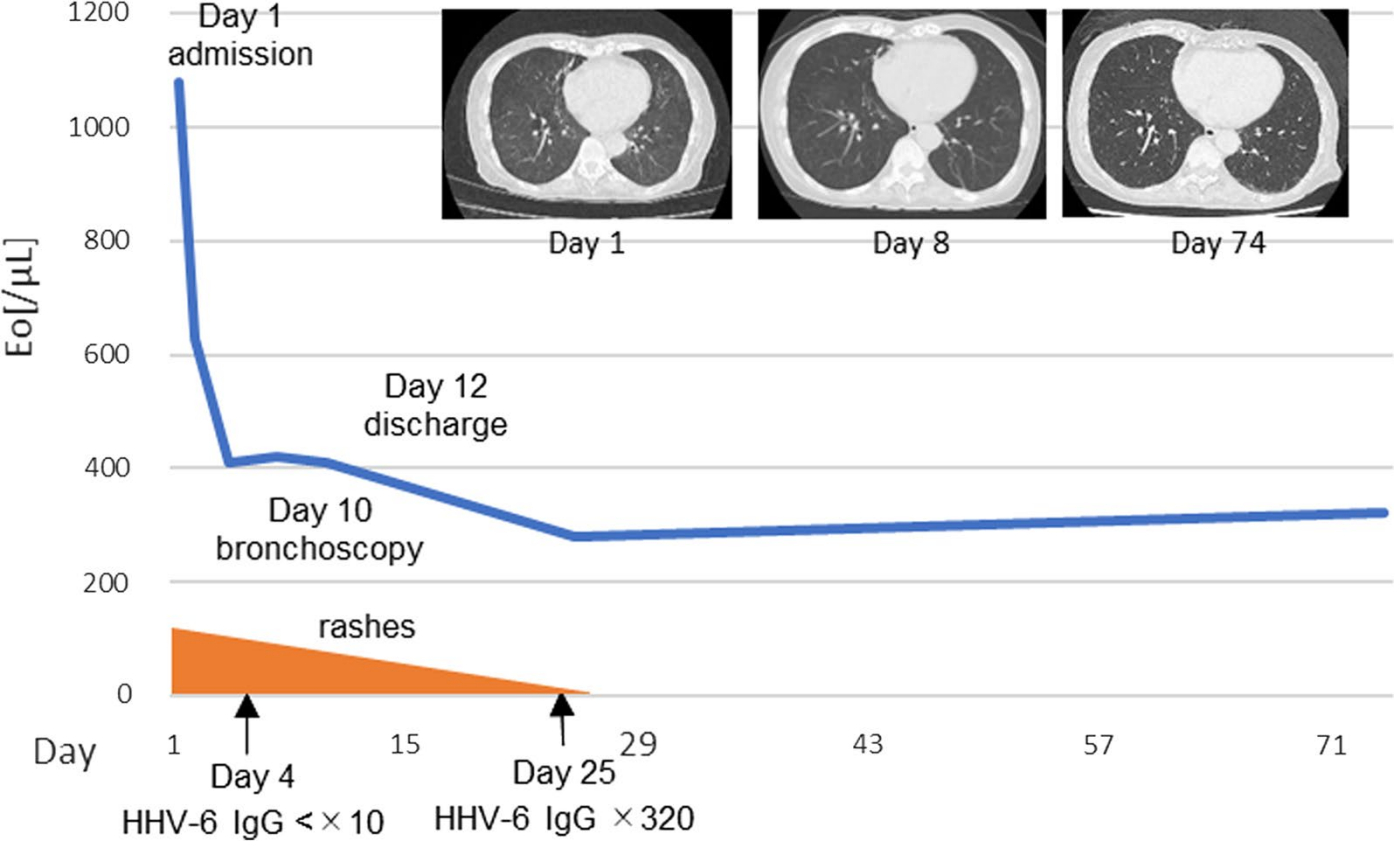
Clinical course

The titer of serum anti-HHV-6 immunoglobulin G (IgG) measured in the blood samples was obtained on hospital day-4, and it showed less than ×10; it increased to ×320 after 3 weeks. Drug-induced lymphocyte stimulation test (DLST) and skin patch test were performed. The skin patch test was positive for minodronic acid hydrate. The DLST for minodronic acid hydrate revealed a slight elevation in the stimulation index (SI 1.7), while both tests for other drugs, including clopidogrel, were negative. Based on these results, we diagnosed her condition as DIHS/DRESS due to minodronic acid hydrate and considered the pulmonary lesions to be related to DIHS/DRESS. Observation without medication was performed because minodronic acid hydrate had already been discontinued, and her fever, cough, and skin rash were improving due to the administration of a small amount of steroid treatment by a family doctor on admission. She was discharged on hospital day-12. Her symptoms and chest CT findings were entirely resolved, and she had no recurrence (Fig. 3).

## Discussion and conclusions

We describe a rare DIHS/DRESS case due to minodronic acid hydrate accompanied by bilateral diffuse ground-glass opacities with ill-defined centrilobular nodules on a chest CT scan. The patient developed an acute rash five years after starting minodronic acid hydrate in the current case, and the clinical symptoms lasted for a prolonged period after discontinuation. She had fever, liver and lung abnormalities, eosinophilia, inguinal lymphadenopathies, and HHV-6 reactivation. Based on the criteria, we diagnosed her condition as DIHS/DRESS [6, 7]. This case suggests two clinical implications.

First, minodronic acid hydrate could be a causative drug of DIHS/DRESS. Previous reports showed that most DIHS/DRESS cases were associated with specific medications, such as anticonvulsants, allopurinol, and sulfonamides [1]. Several other causative drugs, such as anti-hepatitis C virus agents, anticoagulants, and antipyretic medications, have also been reported [8]. In the present case, minodronic acid hydrate was identified as the culprit drug by DLST and skin patch test. To the best of our knowledge, there have been no reports of DIHS/DRESS caused by minodronic acid hydrate. It suggests that we should be aware of DIHS/DRESS even if the disease is not associated with the typical causative drugs.

Second, the CT findings of this case were atypical for DIHS/DRESS. There were diffuse ground-glass opacities with ill-defined centrilobular nodules on a CT scan. DIHS/DRESS could affect the lungs, and a previous review showed that pulmonary lesions occurred in 5% of the cases [9]. The most common pulmonary manifestation is interstitial infiltration and is present in 50% of DIHS/DRESS cases with lung lesions. Acute respiratory distress syndrome and pleural effusion are described in 31% and 22.7% of cases, respectively [2]. Bilateral nodules and centrilobular granular shadows were reported [1, 3, 4]. However, diffuse ground-glass opacities and ill-defined centrilobular nodules were present at the same time in the present case; they are unique and interesting features of this case.

To investigate the pathogenesis of her lung condition, we performed TBLC in the left lower lobe. Pathological findings showed that lymphocyte infiltration was observed in the alveolar walls and fibrinous exudates and floating macrophages in the alveolar lumina. These findings were consistent with drug-induced lung disease and would have reflected the ground-glass opacities and ill-defined centrilobular nodules on a CT scan, respectively.

In addition, we consider her condition as a recovery phase of DIHS/DRESS based on the result of the immunopathological findings of the TBLC samples. Details of the mechanism of DIHS/DRESS are unknown. However, previous studies have shown that CD8^+^ lymphocytes proliferated during the acute phase and CD4^+^ T cells proliferated during the recovery phase in the cases of DIHS/DRESS [10–12]. Other previous literatures have suggested that increased regulatory T cells (Foxp3^+^) might cause the acute phase of DIHS/DRESS, while they subsequently decreased in the recovery phase [13–15]. In the present case, there were more CD4^+^ lymphocytes than CD8^+^ lymphocytes in the lung biopsy specimen, similar to the CD4/CD8 ratio of BALF (6.2). Few Foxp3^+^ lymphocytes were detected in the lung sample, indicating few regulatory T cells. These findings suggest that this case may have been in a recovery period of DIHS/DRESS. This may be the reason for the unique chest CT findings in this case.

In conclusion, we report the first case of DIHS/DRESS caused by minodronic acid hydrate. Lung lesions in DIHS/DRESS can present with bilateral diffuse ground-glass opacities and ill-defined centrilobular nodules on a CT scan during the recovery phase. When skin rashes with multiple organ dysfunction are seen, clinicians should be aware of DIHS/DRESS, even if the patients are not involved with typical DIHS/DRESS-causing drugs.

## Supplementary Information

**Additional file 1: Figure S1**. Low power views of lung biopsy specimens. **A**–**C** Immunohistochemistry of biopsy specimens with CD8 (A), CD4 (B), and Foxp3 (C) stains shows more CD4^+^ lymphocytes than CD8^+^ lymphocytes, while few Foxp3^+^ lymphocytes were observed (× 25). The areas of squares of A, B, and C correspond to Fig. 2D, E, and F, respectively. Scale bar = 400 µm.

## Data Availability

All data are contained within the manuscript.

## Abbreviations

DIHS: Drug-induced hypersensitivity syndrome
DRESS: Drug reaction with eosinophilia and systemic symptoms
HHV-6: Human herpesvirus 6
CT: Computed tomography
IgE: Immunoglobulin E
BALF: Bronchoalveolar lavage fluid
TBLC: Transbronchial lung cryobiopsy
IgG: Immunoglobulin G
DLST: Drug-induced lymphocyte stimulation test
SI: Stimulation index

## References

[1] Shibuya R, Tanizaki H, Nakajima S, Nakajima S, Koyanagi I, Kataok TR, Miyachi Y, Kabashima K (2015). DIHS/DRESS with remarkable eosinophilic pneumonia caused by zonisamide. Acta Derm Venereol.

[2] Taweesedt PT, Nordstrom CW, Stoeckel J, Dumic I (2019). Pulmonary manifestations of drug reaction with eosinophilia and systemic symptoms (DRESS) syndrome: a systematic review. Biomed Res Int.

[3] Lee SP, Kim SH, Kim TH, Sohn JW, Shin DH, Park SS, Yoon HJ (2010). A case of mexiletine-induced hypersensitivity syndrome presenting as eosinophilic pneumonia. J Korean Med Sci.

[4] Sawata T, Bando M, Kogawara H, Nakayama M, Mato N, Yamasawa H, Takeura T, Sugiyama Y (2016). Drug-induced hypersensitivity syndrome accompanied by pulmonary lesions exhibiting centrilobular nodular shadows. Intern Med.

[5] Miyazu D, Kodama N, Yamashita D, Tanaka H, Inoue S, Imakyure O, Hirakawa M, Shuto H, Kataoka Y (2016). DRESS syndrome caused by cross-reactivity between vancomycin and subsequent teicoplanin administration: a case report. Am J Case Rep.

[6] Shiohara T, Iijima M, Ikezawa Z, Hashimoto K (2007). The diagnosis of a DRESS syndrome has been sufficiently established on the basis of typical clinical features and viral reactivations. Br J Dermatol.

[7] Kardaun SH, Sidoroff A, Valeyrie-Allanore L, Halevy S, Davidovici BB, Mockenhaupt M, Roujeau JC (2007). Variability in the clinical pattern of cutaneous side-effects of drugs with systemic symptoms: does a DRESS syndrome really exist?. Br J Dermatol.

[8] Cho YT, Yang CW, Chu CY (2017). Drug reaction with eosinophilia and systemic symptoms (DRESS): an interplay among drugs, viruses, and immune system. Int J Mol Sci.

[9] Cacoub P, Musette P, Descamps V, Meyer O, Speirs C, Finzi L, Roujeau JC (2011). The DRESS syndrome: a literature review. Am J Med.

[10] Picard D, Janela B, Descamps V, Incan MD, Courville P, Jacquot S, Rogez S, Mardivirin L, Teisserenc HM, Toubert A, Benichou J, Joly P, Musette P (2010). Drug reaction with eosinophilia and systemic symptoms (DRESS): a multiorgan antiviral t cell response. Sci Transl Med.

[11] Hanafusa T, Azukizawa H, Matsuura S, Katayama I (2012). The predominant drug-specific T-cell population may switch from cytotoxic T cells to regulatory T cells during the course of anticonvulsant-induced hypersensitivity. J Dermatol Sci.

[12] Hase I, Arakawa H, Sakuma H, Kaneko F, Watanabe Y, Fujiu K, Miyamoto H, Isihi Y (2016). Bronchoscopic investigation of atypical drug-induced hypersensitivity syndrome showing viral lung involvement. Intern Med.

[13] Shiohara T, Mizukawa Y (2019). Drug-induced hypersensitivity syndrome (DiHS)/drug reaction with eosinophilia and systemic symptoms (DRESS): an update in 2019. Allergol Int.

[14] Takahashi R, Kano Y, Yamazaki Y, Kimishima M, Mizukawa Y, Shiohara T (2009). Defective regulatory T cells in patients with severe drug eruptions: timing of the dysfunction is associated with the pathological phenotype and outcome. J Immunol.

[15] Morito H, Ogawa K, Fukumoto T, Kobayashi N, Morii T, Kasai T, Nonomura A, Kishimoto T, Asada H (2014). Increased ratio of FoxP3^+^ regulatory T cells/CD3^+^ T cells in skin lesions in drug-induced hypersensitivity syndrome/drug rash with eosinophilia and systemic symptoms. Clin Exp Dermatol.

