# Supplementary figures and images for: Drug-induced hypersensitivity syndrome caused by minodronic acid hydrate
Source: BMC Pulm Med. 2021 Nov 7;21:350. doi: 10.1186/s12890-021-01709-x (PMC8572576; doi:10.1186/s12890-021-01709-x)

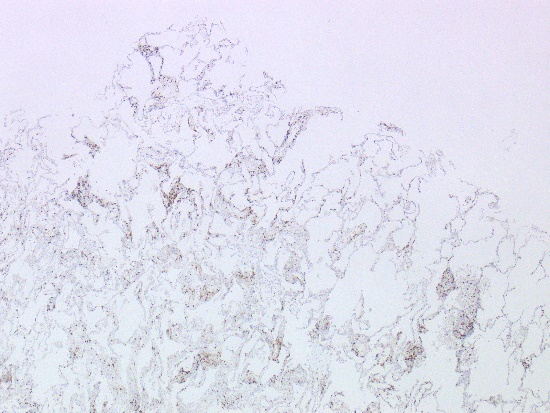


A


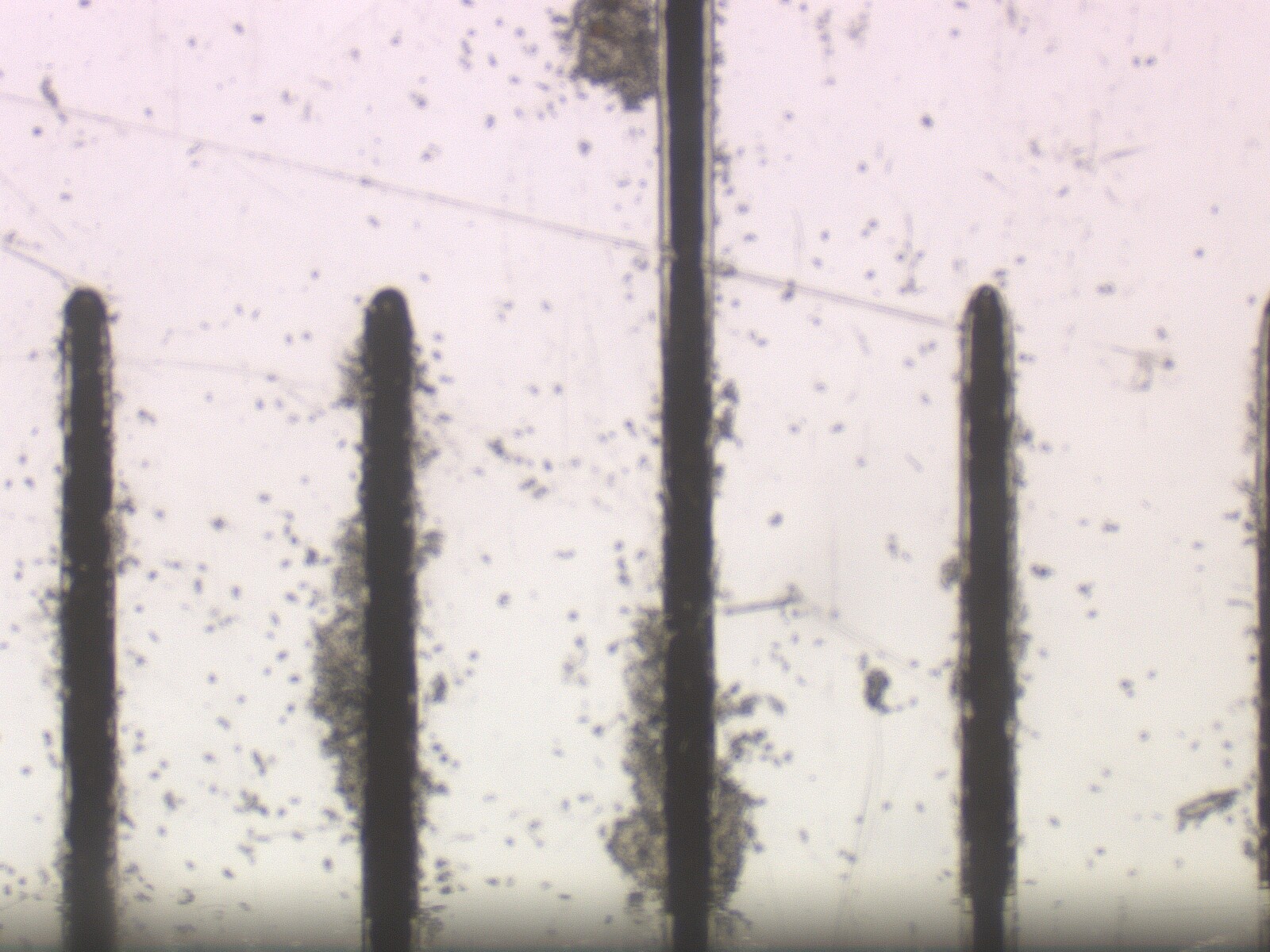


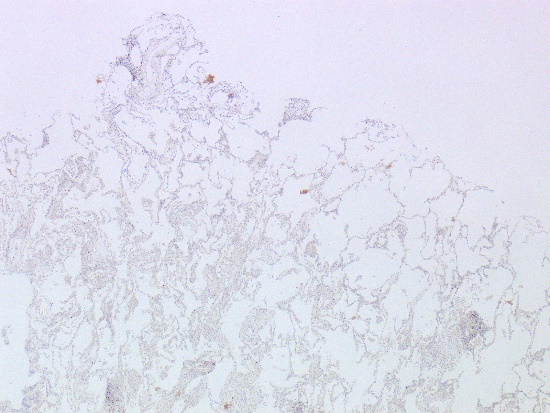


C


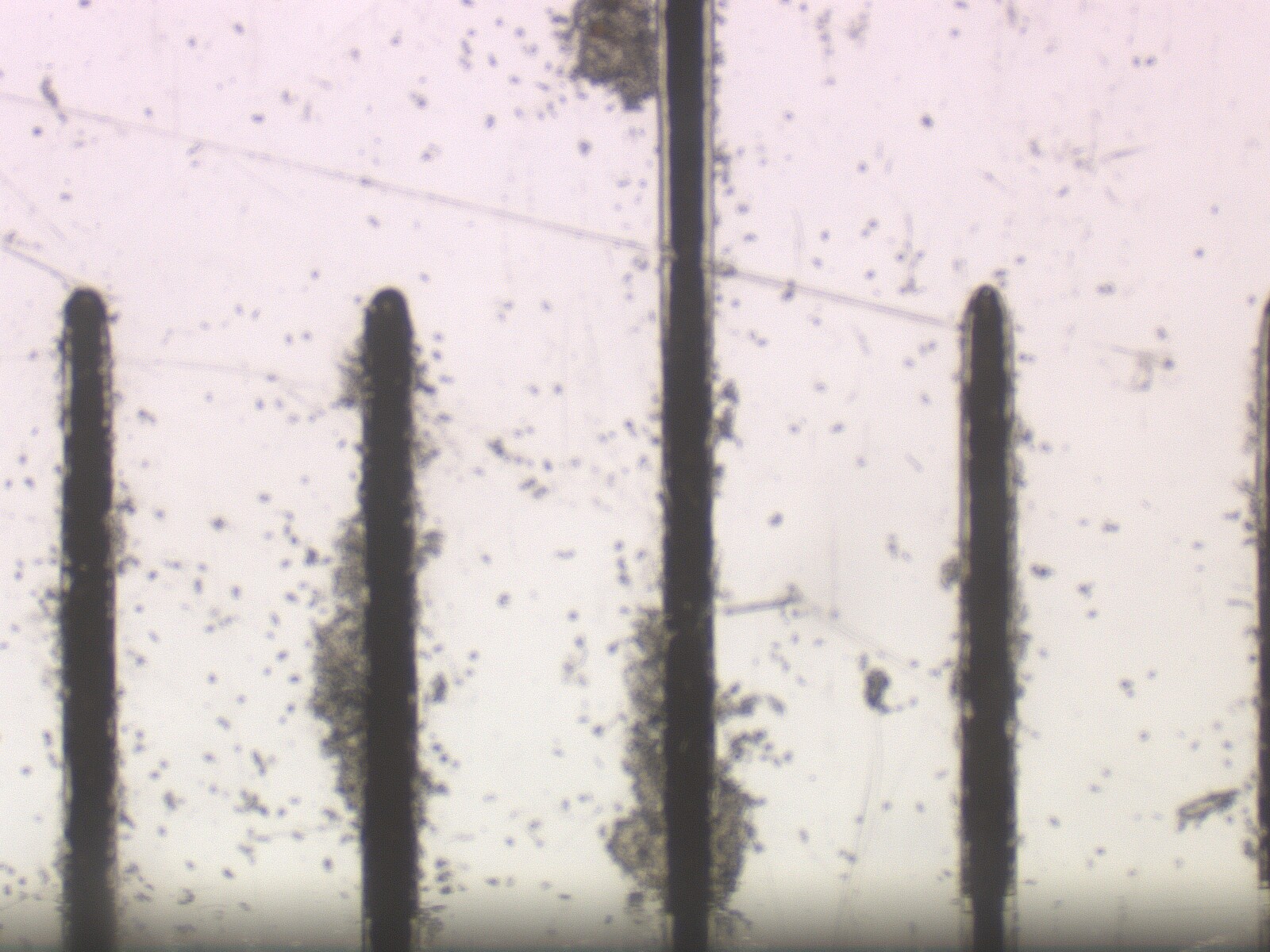


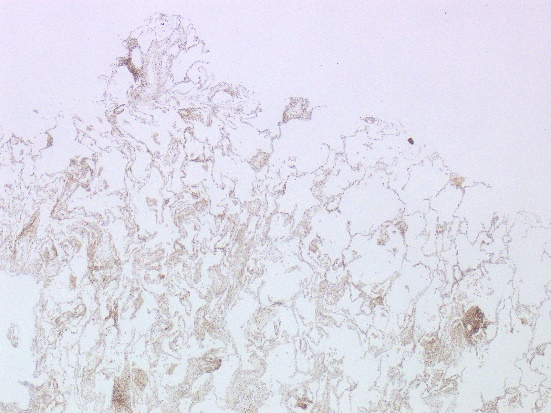


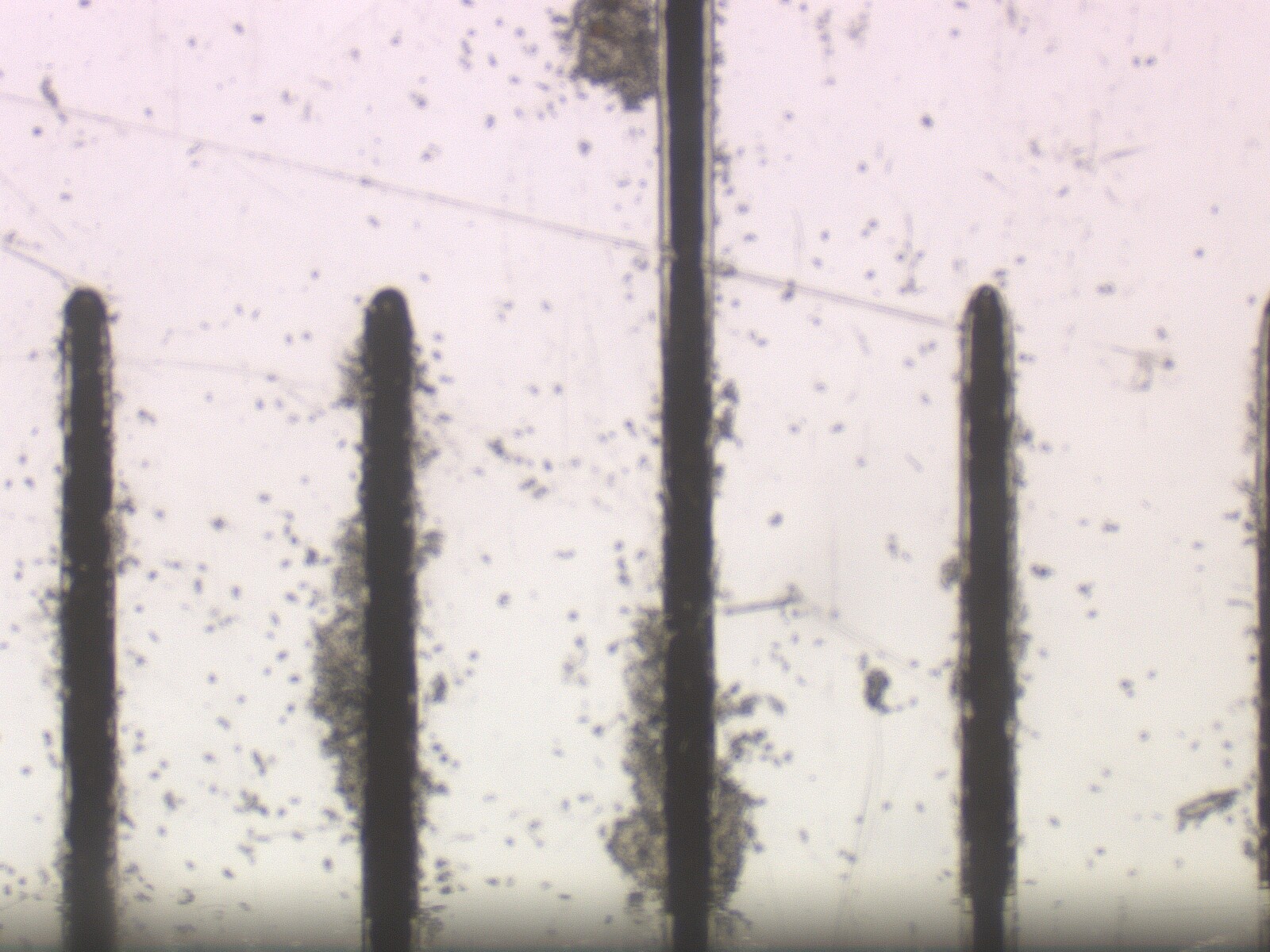


B

Supplement: Supplementary file 1 — Additional file 1: Figure S1. Low power views of lung biopsy specimens. A–C Immunohistochemistry of biopsy specimens with CD8 (A), CD4 (B), and Foxp3 (C) stains shows more CD4+ lymphocytes than CD8+ lymphocytes, while few Foxp3+ lymphocytes were observed (× 25). The areas of squares of A, B, and C correspond to Fig. 2D, E, and F, respectively. Scale bar = 400 µm. [file 12890_2021_1709_MOESM1_ESM.docx]
